# Role of Pirin, an Oxidative Stress Sensor Protein, in Epithelial Carcinogenesis

**DOI:** 10.3390/biology10020116

**Published:** 2021-02-04

**Authors:** Francisco Perez-Dominguez, Diego Carrillo-Beltrán, Rancés Blanco, Juan P. Muñoz, Grettell León-Cruz, Alejandro H. Corvalan, Ulises Urzúa, Gloria M. Calaf, Francisco Aguayo

**Affiliations:** 1 Laboratorio Oncovirología, Programa de Virología, Facultad de Medicina, Instituto de Ciencias Biomédicas, Universidad de Chile, Santiago 8380000, Chile; francisco.perez.d@ug.uchile.cl (F.P.-D.); carrillo.diego.b@gmail.com (D.C.-B.); rancesblanco1976@gmail.com (R.B.); 2 Instituto de Alta Investigación, Universidad de Tarapacá, Arica 1000000, Chile; jp_182_mb@hotmail.com (J.P.M.); gmc24@cumc.columbia.edu (G.M.C.); 3 Centro Universitario del Sur, Laboratorio de Ciencias Fisiológicas, Departamento de Ciencias Básicas para la Salud, Universidad de Guadalajara, Guadalajara 44100, Mexico; grettell.leon@gmail.com; 4 Advanced Center for Chronic Diseases (ACCDIS), Pontificia Universidad Católica de Chile, Santiago 8320000, Chile; acorvalan@uc.cl; 5 Departamento de Oncología Básico Clínica (DOBC), Facultad de Medicina, Universidad de Chile, Santiago 8380000, Chile; uurzua@med.uchile.cl; 6 Center for Radiological Research, Columbia University Medical Center, New York, NY 10032, USA; 7 Instituto de Alta Investigación, Sede Esmeralda, Universidad de Tarapacá, Iquique 1101783, Chile

**Keywords:** pirin, cancer, epithelial

## Abstract

**Simple Summary:**

Pirin is a protein which is detected at low levels in normal tissues. However, it is detected at high levels in multiple cancers, particularly in melanomas, cervical cancer or squamous cell lung carcinomas. Essentially, its role in cancer is related to the host response against factors causing oxidative stress, favoring cell migration and metastasis. Here we summarize the biological functions of Pirin in relation to its role in cancer, suggesting that Pirin is a potential therapeutic target.

**Abstract:**

Pirin is an oxidative stress (OS) sensor belonging to the functionally diverse cupin superfamily of proteins. Pirin is a suggested quercetinase and transcriptional activator of the nuclear factor kappa-light-chain-enhancer of activated B cells (NF-κB) pathway. Its biological role in cancer development remains a novel area of study. This review presents accumulating evidence on the contribution of Pirin in epithelial cancers, involved signaling pathways, and as a suggested therapeutic target. Finally, we propose a model in which Pirin is upregulated by physical, chemical or biological factors involved in OS and cancer development.

## 1. Introduction

The cupin superfamily of proteins is considered to be one of the most functionally diverse protein superfamilies. These proteins are characterized by their conserved β-barrel structure and two distinctive short signature sequence motifs: PGX5HXHX3,4-EX6G and GX5PXGX2HX3N, which contain residues responsible for binding to metal ions. There is also a 15–50 amino acid sequence located between both motifs [1,2,3,4]. Interestingly, a range of metal ions bind to the cupin active site, including nickel, iron, manganese, copper, zinc, and cadmium, accounting for biological functions of cupin proteins [5]. They display diverse enzymatic functions such as dioxygenases, isomerases, oxalate oxidases and non-enzymatic activities such as auxin binding, sucrose binding, seed storage, and transcriptional factor, in Archaea, Eubacteria, and Eukaryota [1,6,7]. Although the focus was initially given to the physiological functions of cupin proteins, new insights reveal this superfamily may be involved in human diseases [8,9,10]. Pirin protein, a cupin superfamily member with a suggested role in cancer, is of particular interest. In this review, we evaluate a proposed role of Pirin in epithelial carcinogenesis, its regulation, and its potential usefulness as a therapeutic target.

## 2. Pirin Structure and Biological Functions

**Figure 1:**
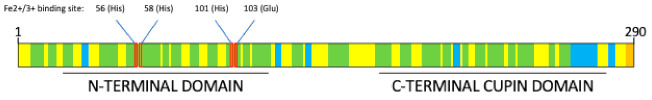
Secondary Pirin structure (*Homo sapiens*). Yellow: Protein Data Bank (PDB) structure known for this area; Green: Beta strands; Blue: Helix; Orange: Turn. Red: Fe2+/3+ binding site. Data extracted from Uniprot Consortium [16].

Pirin is a 32 kDa protein with 290 amino acids showing a dot-like nuclear localization, which is highly conserved among mammals, plants, fungi, and prokaryotes. In *Homo sapiens*, Pirin transcripts are highly expressed in both muscle and cardiac tissues [11]. Winaker’s group cloned the PIR gene for the first time and characterized the expressed protein from an in vitro model [11,12]. The crystal Pirin structure [12] shares characteristics with cupin proteins such as two antiparallel germin-like β-barrel domains, the two distinctive cupin family motifs, a single Fe2+ placed in its N-terminal domain, and a C-terminal domain that contains one α-helix (Figure 1). Curiously, this last domain does not include another metal-binding site found in other cupin proteins [3,11,12]. It was also described that the metal ion grants greater stabilization to Pirin’s crystal structure and explains its biological functions [12]. Regarding its enzymatic function, the initial evidence showed that Pirin is functionally similar to quercetin 2,3-dioxygenase. In fact, both Pirin and quercetin 2,3-dioxygenase use quercetin, a flavonoid widely known for its antioxidant activity in human beings, as a substrate [13,14]. Additionally, carbon monoxide is released during the enzymatic reaction in both cases, and quercetin 2,3-dioxygenase inhibitors are also able to inhibit Pirin activity, suggesting a quercetinase activity of Pirin [13]. In the same way, the Pirin bacteria ortholog Pirin_sm_ interacts with pyruvate dehydrogenase E1 subunit, and consequently inhibits and regulates the catabolism of pyruvate to acetyl-CoA [15].

The second function attributed to Pirin is acting as a transcriptional co-regulator. Initially, it was reported that Pirin interacts with the nuclear factor I/CCAAAT box transcription factor (NF-I) and binds to the hematologic oncogene B-cell lymphoma 3-encoded protein (BCL-3), forming complexes along with nuclear factor kappa-light-chain-enhancer of activated B cells (NF-κB) p50 [11,17,18]. NF-κB is a ubiquitous transcriptional regulator considered central in immune response, apoptosis, inflammation, and oxidative stress (OS) [19,20,21]. More recently, it was described that under oxidizing conditions, Pirin metal structurally changes from Fe2+ (inactive form) to Fe3+ (active form) and functionally enhances the binding affinity to NF-κB p65, which suggests a previously unknown role of Pirin as an iron-dependent OS modulator [22]. However, the Pirin Fe center does not represent its enzymatic active site, but functions as an allosteric control site. Meanwhile, the R-shaped surface loop area of Pirin is structurally modified depending on the metal oxidation state, and thus, the two resulting conformational states, active and inactive, are crucial for Pirin’s ability to regulate NF-κB [22]. In addition, the Fe3+ form of Pirin shows restricted conformational space and electrostatic complementarity which are crucial for NF-kB binding [23,24]. Notably, these redox mediator functions are also observed in plants and prokaryotes in which Pirin-like proteins regulate oxidative pathways and are closely related to cell death [25,26]. Additionally, Pirin is expressed at high levels in the kidney and spleen of transgenic cytosolic superoxide dismutase (Sod1)-deficient mice [27]. All the above findings are summarized in Table 1. 

**Figure 2:**
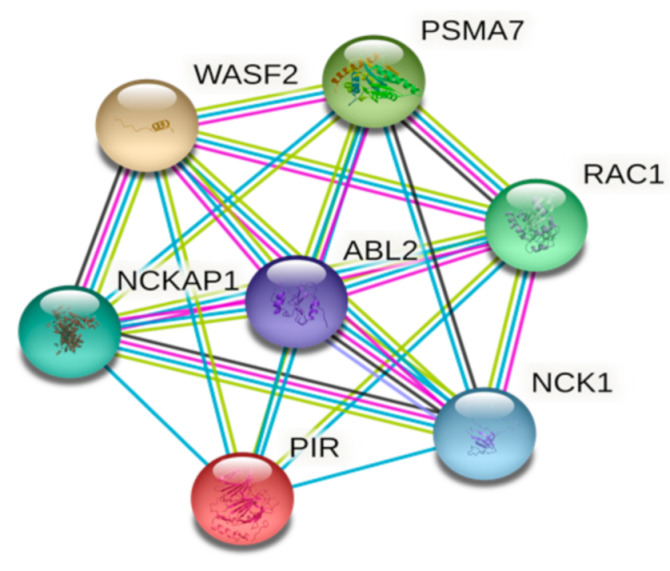
Pirin functional association network. Filled nodes represent proteins with predicted 3D structures. Edges color, Purple: experimentally determined; light blue: association in curated databases; green: co-mentioned in PubMed abstracts (STRING V11.0, full STRING network, confidence 0.900) [39]. Abbreviations: WASF2: Wiskott—Aldrich syndrome protein family member 2; PSMA7: proteasome subunit alpha type-7; RAC1: Ras-related C3 botulinum toxin substrate 1; NCK1: cytoplasmic protein; NCKAP1: Nck-associated protein 1; ABL2: Abelson tyrosine-protein kinase 2.

A bioinformatic analysis demonstrated that Pirin is functionally associated with Wiskott–Aldrich syndrome protein family member 2 (WASF2), Proteasome subunit alpha type-7 (PSMA7), Ras-related C3 botulinum toxin substrate 1 (RAC1), Nck-associated protein 1 (NCKAP1), Cytoplasmic protein (NCK1) and Abelson tyrosine-protein kinase 2 (ABL2) (Figure 2). WASF2 is involved in signal transmission from tyrosine kinase receptors to actin filaments leading to lamellipodia formation [28,29]. PSMA7 participates in the ATP-dependent degradation of ubiquitinated proteins [30]. RAC1 is a GTPase which is involved in a plethora of cellular processes such as cell growth, cytoskeletal reorganization, and protein kinase activation, among others [31,32]. NCKAP1 is part of the WAVE complex that regulates lamellipodia formation through the reorganization of actin filaments, is involved in cell invasion, and promotes cancer cell malignancy [33,34]. NCK1 is an adapter protein which interacts with tyrosine-phosphorylated growth factor receptors, playing a role in DNA damage response by activating downstream effectors. In addition, NCK1 is involved in actin cytoskeletal remodeling, podosome formation and cancer invasion [35,36]. ABL2 is a cytoplasmic tyrosine kinase which coordinates actin remodeling through tyrosine phosphorylation of proteins controlling cytoskeleton dynamics. Taken together, the functional association of Pirin with proteins involved in cytoskeleton reorganization partially explains the link between Pirin overexpression and increased cell migration in some cellular models [37,38]. Further experimental settings are warranted to elucidate the molecular mechanisms involved in these functional associations. 

## 3. Role of Pirin in Cancer Development

Recent investigations have been carried out on the contribution of Pirin in various types of cancer, including epithelial tumors [40] as well as those from the hematopoietic and neurological systems [41,42]. Additionally, Pirin has been reported as a potential molecular target of metastatic suppressors [43]. The following sections summarize the interaction of Pirin with carcinogenic agents and its potential role in epithelial carcinogenesis.

### 3.1. Lung Cancer

Lung cancer is the most commonly diagnosed cancer in women and men worldwide, with tobacco smoke (TS) being the predominant cause [44,45]. It has been reported that Pirin levels are remarkably raised in the airway epithelium of chronic smokers [46]. Likewise, cigarette smoke extracts upregulate Pirin levels in human bronchial epithelial cells in a dose-dependent manner [47]. Such Pirin upregulation induces apoptosis of epithelial cells which may be explained by an interaction between Pirin and NF-κB [47]. Another study showed increased Pirin levels in human small airway epithelial cells exposed to TS, suggesting a potential role of Pirin in TS-associated injury [48]. Accordingly, other studies demonstrated that Pirin is upregulated in bronchial epithelial cells exposed to cigarette smoke extracts and is accompanied by ferritin gene upregulation, a well-known marker of ferroptosis [49]. Furthermore, PIR has been identified as a novel nuclear factor erythroid 2-related factor 2 (NRF2)-modulated gene in the small airway epithelium of healthy smokers [50]. NRF2, an oxidant responding transcription factor highly active in lung cancer cell lines [51,52], can bind to the PIR promoter which contains four functional antioxidant response elements (ARE) [50,53]. This association between NRF2 and PIR may explain a possible OS-regulation in the tobacco smoke-exposed airway epithelial cells [50]. However, NRF2 activation/addiction may be involved in other Pirin-independent activities such as cisplatin resistance in lung cancer cells [54,55]. Additionally, increased Pirin expression is found in alveolar macrophages of mice exposed to TS for 1 to 3 months, suggesting that Pirin may also be involved in macrophage activation [56]. Of note, Pirin levels show a strong correlation with smoking, as well as chronic obstructive pulmonary disease (COPD) [57], which frequently progresses to lung cancer. Moreover, particulate matter in outdoor air pollution, considered to be a potent oxidative agent and a significant risk factor for respiratory diseases and lung cancer [58], is associated with Pirin overexpression in human respiratory fibroblasts [57]. Additionally, the telomerase RNA component (TERC) encoding gene, upregulated in non-small cell lung carcinoma (NSCLCs) [59], was found to be regulated by Pirin [60]. Taken together, the above findings lead us to suggest a potential role of Pirin in lung cancer, although additional studies are necessary to confirm these observations. 

### 3.2. Cervical Cancer

Cervical cancer is the fourth most prevalent cancer among women, causing a total of 311,000 deaths in 2018 [61]. The persistent infection of high-risk human papillomavirus (HPV) is considered a necessary condition for cervical cancer development [62]. We demonstrated for the first time that Pirin is expressed in a HPV load-dependent manner in cervical cancer cells [63]. Additionally, it was demonstrated that PIR knockdown increases E-cadherin levels and reduces Slug, zinc finger E-box-binding homeobox protein (ZEB) and Snail in cervical cancer cells, suggesting its contribution to epithelial–mesenchymal transition (EMT) and cell migration [63]. However, there is an interaction between Pirin, B-cell lymphoma 3-encoded protein (BCL-3), and Slug in melanoma cells, whereas in cervical cancer cells Pirin induces EMT by decreasing E-cadherin expression independent of BCL-3-Slug signaling [38,64]. Interestingly, we found that Pirin levels are increased by HPV16 E6 and E7 oncoproteins in infected cervical cancer cells, unlike HPV-negative cells [63]. Moreover, curcumin, a common food additive and well-known antioxidant agent [65,66], has been reported to interfere with EMT in HeLa cells (HPV-18) and breast cancer cell lines [67,68,69]. Notably, we demonstrated that curcumin decreases Pirin levels and reduces EMT and cell migration, suggesting a novel Pirin-dependent mechanism wherein curcumin rescues cervical cancer cells from EMT [70]. It has been shown that basal Pirin expression is strongly dependent on NRF2, due to its crucial interaction with an ARE site located in a short region downstream of the transcription start site (TSS) [71]. Considering that Pirin is a NF-κB activator, we hypothesize that Pirin may act as a mediator between NRF2 and NF-κB in cervical cancer cells [71]. 

### 3.3. Skin Cancer

Skin cancer is the most common cancer in the United States when non-melanoma carcinomas are included in the registries [72], and approximately 100,000 new melanoma cases are diagnosed every year [73]. In this type of skin cancer, a relevant role in cell migration and progression was identified for Pirin [38,74]. Miyasaki et al. identified high expression of Pirin in melanoma cell lines. In addition to this discovery, they found that a small molecule named Triphenyl compound A (TPhA) blocks the interaction between Pirin and BCL-3, inhibiting cell migration [38]. It was also established that Pirin localizes in the nucleus or cytoplasm, depending on the stage of melanoma progression. In fact, a significant proportion of cytoplasmatic Pirin was found in metastatic melanoma cells compared to primary melanoma cells, suggesting that this pattern of Pirin localization may represent a cancer progression biomarker [74]. Moreover, the same group identified that Pirin is barely expressed in mature nevus samples compared to the high levels found in primary and metastatic melanomas. By inducing PIR knockdown, it was also found that metastatic melanoma cells change both morphology and size, which was compatible with a senescent phenotype. Altogether, these results suggest that Pirin contributes to the metastatic properties of melanoma cells [75]. 

It has been extensively reported that ultraviolet (UV) radiation is a major risk factor for melanomas [76]. It is important to note that UV radiation, specifically UV-A, induces NRF2 nuclear translocation and accumulation and may activate NRF2-controlled proteins in human skin cells [77]. Moreover, UV-A radiation promotes reactive oxygen species (ROS) production and subsequently triggers the activation of NRF2 in melanocytes [78]. Even though the mentioned findings consider Pirin regulator-NRF2 signaling pathways, there are still no studies that specifically assess the effect of UV radiation in Pirin levels. In miR-155-overexpressing melanocytic cancer cells (CL16-miR-155), Pirin shows less intensity stain than controls by immunohistochemistry (IHC) assays [79]. Since miR-155 overexpression may inhibit tumor dissemination, extravasation, and colonization, Pirin may mediate, whether directly or indirectly, metastasis development [79,80]. In addition to these findings, after treatment with Hsc025, a novel molecule that improves hepatic fibrosis-degree and stimulates wound healing, Pirin levels modestly increase in fibroblasts [81,82]. However, PIR knockdown significantly counteracts the effect of Hsc025 on fibroblast migration, suggesting that Pirin may be a key intermediary in this process [82]. More recently, another novel compound known as bisamide (CCT251236) was identified as a potent ligand of Pirin [83]. It is important to note that these molecules do not directly interact; in contrast, bisamide amide groups and Pirin Fe bind through a water-mediated interaction. Additionally, there is consistent evidence that bisamide modulates the expression of the transcription factor HSF1 and subsequently inhibits the migration of human melanoma cells WM266.4; however, Pirin participation remains unclear [83]. In light of these findings, it was recently demonstrated that both CCG-222740 and CCG-257081, which are compounds structurally similar to CCT251236 and possible inhibitors of metastatic and fibrotic signals, are also able to bind to Pirin [43]. Moreover, they show that such CCG compounds may disrupt Pirin expression, and subsequently interfere with MRTF/SRF/DNA signaling, which has been associated with melanoma metastasis [43].

### 3.4. Breast Cancer

In 2018, breast cancer was the second most prevalent cancer and the second most common cause of death among women in the United States [44,84]. Several genes have been shown to alter their expression pattern in human breast tumors, and some of these may have a role in predicting clinical prognosis [85,86]. One of these candidates includes the potential participation of the PIR gene [87]. Based on a previous report in which Pirin expression significantly varied between metastasis patients and non-metastasis patients [87], Shubbar et al. showed that Pirin levels in normal breast cells do not differ from invasive breast cancer samples. Nevertheless, Pirin levels are highly correlated with positive axillary lymph nodes status, suggesting a connection between high Pirin levels and local metastasis [88]. Furthermore, PIR knockdown resulted in a noticeably reduced cell proliferation rate in breast cancer cells and decreased xenograft tumor growth in mice [89]. In addition, Pirin mediates breast tumorigenesis by promoting E2F1 expression, a key cell cycle regulator that is abnormally active in malignant tumors [89,90,91]. In fact, Pirin binds to the 3′-terminal region of the E2F1 promoter and subsequently facilitates G1 to S phase transition in breast cancer cells [89]. 

### 3.5. Head and Neck and Gastrointestinal Cancers

**Figure 3:**
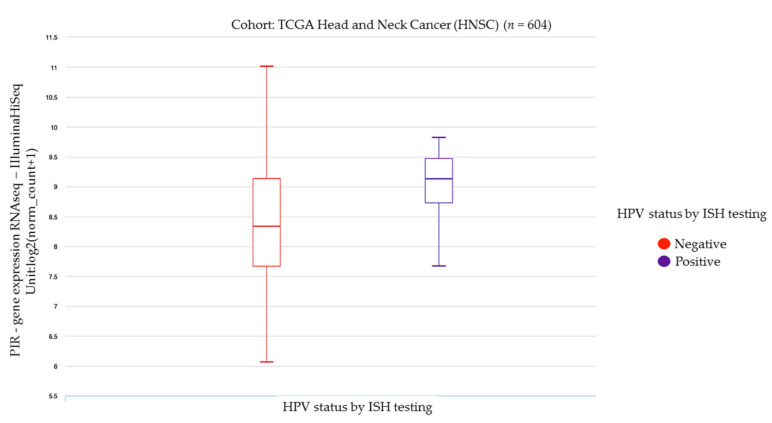
PIR transcripts expression in head and neck cancers (TCGA, *n* = 604) stratified by ISH testing for human papillomavirus (*p* = 0.02028, Welch’s *t*-test). Raw data were extracted from University of California, Santa Cruz (ena.ucsc.edu). UCSC Xena functional genomics explorer (https://xenabrowser.net) [96].

Other epithelial cancers such as oral, pancreas, biliary tract, and colorectal carcinomas have also been linked with changes in Pirin expression. First, we demonstrated that Pirin is overexpressed in oral cells expressing HPV16 E6 and E7 oncoproteins [63]. To confirm these results in clinical specimens, we evaluated the relationship between PIR gene expression and HPV status in head and neck carcinomas from The Cancer Genome Atlas (TCGA) consortium [92]. This analysis showed a statistically significant increase in PIR gene expression in HPV positive cases (evaluated by in situ hybridization) when compared with HPV negative ones (*p* = 0.02028, Welch’s *t*-test) (Figure 3). Furthermore, both epidermal growth factor receptor/mitogen-activated protein kinase kinase/extracellular signal-regulated kinase (EGFR/MEK/ERK) and phosphoinositide 3-kinase/protein kinase B (PI3K/AKT) pathways may be involved in the activation of Pirin by HPV-E7 oncoprotein in oral cells [63], which is in line with a previous report on transformed rat fibroblasts [93]. More recently, we demonstrated that EGFR/PI3K/AKT1/NRF2 signaling promotes Pirin-dependent canonical NF-κB activation, which, in turn, promotes oral cell migration [37]. Altogether, this strongly suggests a novel carcinogenic mechanism by which Pirin is involved in oral cancer, although the clinical consequences remain to be determined. Similar to the aforementioned findings in lung cells [50], Pirin is strictly regulated by NRF2 in pancreas cancer cells [94]. Moreover, high Pirin levels are associated with a reduced survival probability in cholangiocarcinoma patients, suggesting that Pirin is a plausible prognostic biomarker [95].

### 3.6. Non-Epithelial Cancers

**Figure 4:**
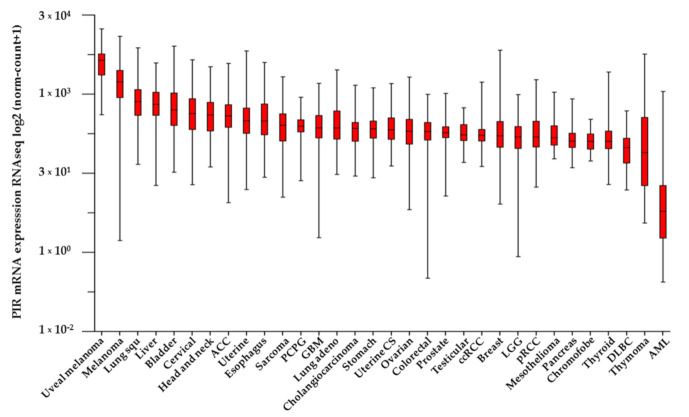
PIR transcript expression in human cancer (TCGA, *n* = 10071). *p* < 0.0001, Welch’s ANOVA test. Raw data were extracted from University of California, Santa Cruz (ena.ucsc.edu). UCSC Xena functional genomics explorer (https://xenabrowser.net) [96].

While Pirin has been widely studied in epithelial cancers, other studies have proposed it as a promising prognostic marker as well as an anti-proliferative therapeutic target of curcumin in hematopoietic and neurological tumors [42,97]. Pirin is downregulated in acute myeloid leukemias, and indeed, its ablation may impair myeloid differentiation and maturation in both humans and mice [41]. Furthermore, Pirin is associated with contrasting high levels of peripheral white blood cells in B-precursor acute lymphoblastic leukemia, which entails a distinctive prognosis in leukemia patients [98,99]. In Figure 4, PIR gene expression in 10,071 patients with epithelial and non-epithelial tumors extracted from TCGA consortium is shown [92]. As observed, PIR transcripts are differentially detected in tumors from a different origin (*p* < 0.0001, Welch’s ANOVA test). Additionally, PIR is highly expressed in tumors including melanomas, lung, cervical or head and neck cancers among others when compared with leukemia, thymoma or diffuse large B-cell lymphoma (DOBC).

## 4. Conclusions

**Figure 5:**
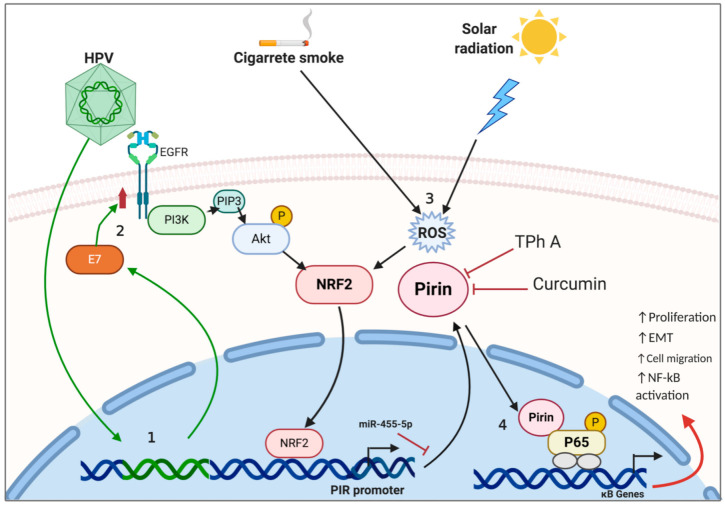
Model of Pirin-mediated tumor activation by viruses or environmental factors in epithelial cells. (1) HPV integrates into the host genome, overexpressing viral oncogenes (i.e., E7). (2) E7 promotes EGFR/PI3K/AKT activation and NRF2 recruitment into the PIR promoter leading to PIR expression. (3) Environmental factors such as cigarette smoke or UV radiation through reactive oxygen species (ROS) generation promote NRF2 recruitment into the PIR promoter. (4) Overexpressed Pirin (Fe3+ status) binds nuclear p65 promoting expression of κB genes. EGFR (epidermal growth factor receptor), PI3K (phosphoinositide 3-kinase), NRF2 (nuclear factor erythroid 2-related factor 2), ROS (reactive oxygen species), NF-κB (nuclear factor kappa B), EMT (epithelial–mesenchymal transition).

Pirin is an established OS sensor and part of the functionally diverse cupin superfamily. Similar to other cupin proteins, Pirin shows enzymatic properties and acts as a nuclear transcriptional regulator. However, its potential oncogenic activity has been a growing topic of discussion in the past years. Recent findings have shown that Pirin plays a role in the development of cancer in epithelial lung, skin, cervical, and oral tumors. Diverse factors (such as environmental and viral) promote ROS increase, consequently raising Pirin levels for activating NF-κB, cell proliferation, cell migration and EMT. In addition, HPV E7 oncoprotein promotes EGFR/PI3K/AKT/NRF2 pathway activation, leading to NRF2 recruitment in the PIR promoter, in turn increasing Pirin expression (Table 2 and Figure 5). This suggests a key participation of Pirin in cancer promotion and progression, although additional experimental approaches, including animal models, for testing Pirin-mediated tumorigenesis are warranted. Thus, this accumulating evidence provides auspicious signs of Pirin as a significant potential biomarker or therapeutic target in the years to come.

## Figures and Tables

**Table 1:** Biological functions of Pirin.

Model	Function	Description	Reference
Human	Enzymatic	Quercetinase activity	[13]
Prokaryote	Co-Enzymatic	Inhibition of acetyl-CoA catabolism	[15]
Human	Transcriptional regulator	Interaction with NF-I/BCL-3/NF-κB p50	[11]
Human	Transcriptional regulator/Redox sensor	Binding to NF-κB p65 in oxidative conditions	[22]
Human	Transcriptional regulator	Fe active form favors its binding and regulation to NF-κB/DNA	[23,24]
Plants	Transcriptional regulator/Redox sensor	Regulation of oxidative pathways and cell death and redox sensor	[26]
Animal	Redox sensor	Activation in superoxide dismutase (Sod1)-deficient mice	[27]

Abbreviations: NF-I: nuclear factor I; BCL-3: B-cell lymphoma 3-encoded protein; NF-κB: nuclear factor kappa B.

**Table 2:** Biological and chemical factors involved in Pirin regulation in human cancers

Cancer	Factors	Regulation	Comments	Ref.
Lung	TS	Activation	Pirin levels are increased in airway epithelium of chronic smokers	[46]
	TS	Activation	Pirin overexpression occurs in a dose-dependent manner	[47]
	TS	Activation	Interaction with NF-κB resulting in a pro-apoptotic response	[47]
	TS	Activation	Pirin overexpression is accompanied by ferroptosis markers upregulation	[49]
	TS	Activation	Interaction with NRF2 in smoke-exposed airway epithelial cells	[50]
Cervical	E7 (HPV16)	Activation	Pirin regulates EMT and migration by interacting with NF-κB	[63]
	Curcumin	Suppression	Curcumin decreases Pirin expression, and consequently EMT and cell migration	[70]
Skin	TPh A	Suppression	Interferes the Pirin interaction with BCL-3, and consequently inhibits cell migration	[38]
	miR-155	Suppression	Pirin may mediate metastasis development	[79]
	CCG	Suppression	Inhibition of carcinogenic signaling pathways	[43]
Oral	E7 (HPV16)	Activation	EGFR/MEK/ERK and PI3K/AKT pathways are involved in Pirin activation by HPV16 E7	[63]
	E7 (HPV16)	Activation	Upregulation of c-Rel and p65 through an interplay with Pirin, promotes cell migration and EMT	[37]

Abbreviations: TS: tobacco smoke; NF-κB: nuclear factor kappa B; NRF2: nuclear factor erythroid 2-related factor 2; BCL-3: B-cell lymphoma 3-encoded protein; EMT: epithelial–mesenchymal transition; HPV: human papillomavirus; Slug: zinc finger protein; ZEB: zinc finger E-box-binding homeobox protein; EGFR: epidermal growth factor receptor; MEK: mitogen-activated protein kinase kinase; ERK: extracellular signal-regulated kinase; PI3K: phosphoinositide 3-kinase; AKT: protein kinase B.

## Data Availability

The data supporting Figure 3 and Figure 4 were obtained from The Cancer Genome Atlas (TCGA) consortium. This information is available in https://xenabrowser.net.

## References

[1] Dunwell J.M. (1998). Cupins: A new superfamily of functionally diverse proteins that include germins and plant storage proteins. Biotechnol. Genet. Eng. Rev..

[2] Woo E.J., Dunwell J.M., Goodenough P.W., Marvier A.C., Pickersgill R.W. (2000). Germin is a manganese containing homohexamer with oxalate oxidase and superoxide dismutase activities. Nat. Struct. Biol..

[3] Dunwell J.M., Purvis A., Khuri S. (2004). Cupins: The most functionally diverse protein superfamily?. Phytochemistry.

[4] Clissold P.M., Ponting C. (2001). JmjC: Cupin metalloenzyme-like domains in jumonji, hairless and phospholipase A2β. Trends Biochem. Sci..

[5] Agarwal G., Rajavel M., Gopal B., Srinivasan N. (2009). Structure-Based Phylogeny as a Diagnostic for Functional Characterization of Proteins with a Cupin Fold. PLoS ONE.

[6] Dunwell J.M., Culham A., Carter C.E., Sosa-Aguirre C.R., Goodenough P.W. (2001). Evolution of functional diversity in the cupin superfamily. Trends Biochem. Sci..

[7] Dunwell J.M., Gane P.J. (1998). Microbial Relatives of Seed Storage Proteins: Conservation of Motifs in a Functionally Diverse Superfamily of Enzymes. J. Mol. Evol..

[8] Sarkar B., Kulharia M., Mantha A.K. (2017). Understanding human thiol dioxygenase enzymes: Structure to function, and biology to pathology. Int. J. Exp. Pathol..

[9] Schmidt H.R., Zheng S., Gurpinar E., Koehl A., Manglik A.K.A., Kruse A.C. (2016). Crystal structure of the human σ1 receptor. Nature.

[10] Uekita T., Gotoh I., Kinoshita T., Itoh Y., Sato H., Shiomi T., Okada Y., Seiki M. (2004). Membrane-type 1 Matrix Metalloproteinase Cytoplasmic Tail-binding Protein-1 Is a New Member of the Cupin Superfamily. A possible multifunctional protein acting as an invasion suppressor down-regulated in tumors. J. Biol. Chem..

[11] Wendler W.M.F., Kremmer E., Förster R., Winnacker E.-L. (1997). Identification of Pirin, a Novel Highly Conserved Nuclear Protein. J. Biol. Chem..

[12] Pang H., Bartlam M., Zeng Q., Miyatake H., Hisano T., Miki K., Wong L.L., Gao G.F., Rao Z. (2004). Crystal Structure of Human Pirin. J. Biol. Chem..

[13] Adams M., Jia Z. (2005). Structural and Biochemical Analysis Reveal Pirins to Possess Quercetinase Activity. J. Biol. Chem..

[14] Liu H., Zhang L., Lu S. (2012). Evaluation of Antioxidant and Immunity Activities of Quercetin in Isoproterenol-Treated Rats. Molecules.

[15] Soo P.-C., Horng Y.-T., Lai M.-J., Wei J.-R., Hsieh S.-C., Chang Y.-L., Tsai Y.-H., Lai H.-C. (2006). Pirin Regulates Pyruvate Catabolism by Interacting with the Pyruvate Dehydrogenase E1 Subunit and Modulating Pyruvate Dehydrogenase Activity. J. Bacteriol..

[16] The UniProt Consortium (2019). UniProt: A worldwide hub of protein knowledge. Nucleic Acids Res..

[17] Dechend R., Hirano F., Lehmann K., Heissmeyer V., Ansieau S., Wulczyn F.G., Scheidereit C., Leutz A. (1999). The Bcl-3 oncoprotein acts as a bridging factor between NF-κB/Rel and nuclear co-regulators. Oncogene.

[18] Maldonado V., Melendez-Zajgla J. (2011). Role of Bcl-3 in solid tumors. Mol. Cancer.

[19] Li Q., Verma I.M. (2002). NF-κB regulation in the immune system. Nat. Rev. Immunol..

[20] Sonenshein G.E. (1997). Rel/NF-κB transcription factors and the control of apoptosis. Semin. Cancer Biol..

[21] DiDonato J.A., Mercurio F., Karin M. (2012). NF-κB and the link between inflammation and cancer. Immunol. Rev..

[22] Liu F., Rehmani I., Esaki S., Fu R., Chen L., De Serrano V., Liu A. (2013). Pirin is an iron-dependent redox regulator of NF-B. Proc. Natl. Acad. Sci. USA.

[23] Barman A., Hamelberg D. (2016). Fe(II)/Fe(III) Redox Process Can Significantly Modulate the Conformational Dynamics and Electrostatics of Pirin in NF-κB Regulation. ACS Omega.

[24] Adeniran C., Hamelberg D. (2017). Redox-Specific Allosteric Modulation of the Conformational Dynamics of κB DNA by Pirin in the NF-κB Supramolecular Complex. Biochemistry.

[25] Talà A., Damiano F., Gallo G., Pinatel E.M., Calcagnile M., Testini M., Fico D., Rizzo D., Sutera A., Renzone G. (2018). Pirin: A novel redox-sensitive modulator of primary and secondary metabolism in Streptomyces. Metab. Eng..

[26] Orzáez D., De Jong A.J., Woltering E.J. (2001). A tomato homologue of the human protein PIRIN is induced during programmed cell death. Plant Mol. Biol..

[27] Brzóska K., Stępkowski T.M., Kruszewski M. (2011). Putative proto-oncogene Pir expression is significantly up-regulated in the spleen and kidney of cytosolic superoxide dismutase-deficient mice. Redox Rep..

[28] Chen Z., Borek D., Padrick S.B., Gomez T.S., Metlagel Z., Ismail A.M., Umetani J., Billadeau D.D., Otwinowski Z., Rosen M.K. (2010). Structure and control of the actin regulatory WAVE complex. Nature.

[29] Takenawa T., Miki H. (2001). WASP and WAVE family proteins: Key molecules for rapid rearrangement of cortical actin filaments and cell movement. J. Cell Sci..

[30] Du H., Huang X., Wang S., Wu Y., Xu W., Li M. (2009). PSMA7, a potential biomarker of diseases. Protein Pept. Lett..

[31] Klooster J.P.T., Leeuwen I.V., Scheres N., Anthony E.C., Hordijk P.L. (2007). Rac1-induced cell migration requires membrane recruitment of the nuclear oncogene SET. EMBO J..

[32] Fukata M., Watanabe T., Noritake J., Nakagawa M., Yamaga M., Kuroda S., Matsuura Y., Iwamatsu A., Perez F., Kaibuchi K. (2002). Rac1 and Cdc42 Capture Microtubules through IQGAP1 and CLIP-170. Cell.

[33] Teng Y., Qin H., Bahassan A., Bendzunas N.G., Kennedy E.J., Cowell J.K. (2016). The WASF3–NCKAP1–CYFIP1 Complex Is Essential for Breast Cancer Metastasis. Cancer Res..

[34] Eden S., Rohatgi R., Podtelejnikov A.V., Mann M., Kirschner M.W. (2002). Mechanism of regulation of WAVE1-induced actin nucleation by Rac1 and Nck. Nature.

[35] Chaki S.P., Barhoumi R., Rivera G.M. (2019). Nck adapter proteins promote podosome biogenesis facilitating extracellular matrix degradation and cancer invasion. Cancer Med..

[36] Buvall L., Rashmi P., Lopez-Rivera E., Andreeva S., Weins A., Wallentin H., Greka A., Mundel P. (2013). Proteasomal degradation of Nck1 but not Nck2 regulates RhoA activation and actin dynamics. Nat. Commun..

[37] Carrillo D., Guerrero N., Muñoz J.P., Aedo-Agulera V., Tapia J.C., León O., Calaf G.M., Corvalán A., Boccardo E., Aguayo F. (2020). Human papillomavirus E7 promotes EGFR/PI3K/Akt/NRF2 signaling pathway contributing to PIR/NF-kB activation in oral cancer cells. Cancers.

[38] Miyazaki I., Simizu S., Okumura H., Takagi S., Osada H. (2010). A small-molecule inhibitor shows that pirin regulates migration of melanoma cells. Nat. Chem. Biol..

[39] Szklarczyk D., Gable A.L., Lyon D., Junge A., Wyder S., Huerta-Cepas J., Simonovic M., Doncheva N.T., Morris J.H., Bork P. (2019). STRING v11: Protein–protein association networks with increased coverage, supporting functional discovery in genome-wide experimental datasets. Nucleic Acids Res..

[40] Yoshikawa R., Yanagi H., Hashimoto-Tamaoki T., Morinaga T., Nakano Y., Noda M., Fujiwara Y., Okamura H., Yamamura T. (2004). Gene expression in response to anti-tumour intervention by polysaccharide-K (PSK) in colorectal carcinoma cells. Oncol. Rep..

[41] Licciulli S., Cambiaghi V., Scafetta G., Gruszka A.M., Alcalay M. (2009). Pirin downregulation is a feature of AML and leads to impairment of terminal myeloid differentiation. Leukemia.

[42] Jungk C., Mock A., Exner J., Geisenberger C., Warta R., Capper D., Abdollahi A., Friauf S., Lahrmann B., Grabe N. (2016). Spatial transcriptome analysis reveals Notch pathway-associated prognostic markers in IDH1 wild-type glioblastoma involving the subventricular zone. BMC Med..

[43] Lisabeth E.M., Kahl D., Gopallawa I., Haynes S.E., Misek S.A., Campbell P.L., Dexheimer T.S., Khanna D., Fox D.A., Jin X. (2019). Identification of Pirin as a Molecular Target of the CCG-1423/CCG-203971 Series of Antifibrotic and Antimetastatic Compounds. ACS Pharmacol. Transl. Sci..

[44] Bray F., Ferlay J., Soerjomataram I., Siegel R.L., Torre L.A., Jemal A. (2018). Global cancer statistics 2018: GLOBOCAN estimates of incidence and mortality worldwide for 36 cancers in 185 countries. CA Cancer J. Clin..

[45] Newcomb P.A., Carbone P.P. (1992). The health consequences of smoking: Cancer. Med. Clin. N. Am..

[46] Spira A.E., Beane J., Shah V., Liu G., Schembri F., Yang X., Palma J., Brody J.S. (2004). Effects of cigarette smoke on the human airway epithelial cell transcriptome. Proc. Natl. Acad. Sci. USA.

[47] Gelbman B.D., Heguy A., O’Connor T.P., Zabner J., Crystal R.G. (2007). Upregulation of pirin expression by chronic cigarette smoking is associated with bronchial epithelial cell apoptosis. Respir. Res..

[48] Mercer B.A., Lemaitre V., Powell C.A., D’Armiento J. (2006). The Epithelial Cell in Lung Health and Emphysema Pathogenesis. Curr. Respir. Med. Rev..

[49] Park E.-J., Park Y.-J., Lee S.J., Lee K., Yoon C. (2019). Whole cigarette smoke condensates induce ferroptosis in human bronchial epithelial cells. Toxicol. Lett..

[50] Hübner R.-H., Schwartz J.D., De B.P., Ferris B., Omberg L., Mezey J.G., Hackett N.R., Crystal R.G. (2009). Coordinate Control of Expression of Nrf2-Modulated Genes in the Human Small Airway Epithelium Is Highly Responsive to Cigarette Smoking. Mol. Med..

[51] Itoha K., Chibabc T., Takahashia S., Ishiia T., Igarashia K., Katoha Y., Oyaked T., Hayashid N., Satohe K., Hatayamae I. (1997). An Nrf2/Small Maf Heterodimer Mediates the Induction of Phase II Detoxifying Enzyme Genes through Antioxidant Response Elements. Biochem. Biophys. Res. Commun..

[52] Ohta T., Iijima K., Miyamoto M., Nakahara I., Tanaka H., Ohtsuji M., Suzuki T., Kobayashi A., Yokota J., Sakiyama T. (2008). Loss of Keap1 Function Activates Nrf2 and Provides Advantages for Lung Cancer Cell Growth. Cancer Res..

[53] Chorley B.N., Campbell M.R., Wang X., Karaca M., Sambandan D., Bangura F., Xue P., Pi J., Kleeberger S.R., Bell D.A. (2012). Identification of novel NRF2-regulated genes by ChIP-Seq: Influence on retinoid X receptor alpha. Nucleic Acids Res..

[54] Silva M.M., Rocha C.R.R., Kinker G.S., Pelegrini A.L., Menck C.F.M. (2019). The balance between NRF2/GSH antioxidant mediated pathway and DNA repair modulates cisplatin resistance in lung cancer cells. Sci. Rep..

[55] Hammad A., Namani A., Elshaer M., Wang X.J., Tang X. (2019). “NRF2 addiction” in lung cancer cells and its impact on cancer therapy. Cancer Lett..

[56] Woodruff P.G., Ellwanger A., Solon M., Cambier C.J., Pinkerton K.E., Koth L.L. (2009). Alveolar Macrophage Recruitment and Activation by Chronic Second Hand Smoke Exposure in Mice. COPD.

[57] Tien C.-P., Chen C.-H., Lin W.-Y., Liu C.-S., Liu K.-J., Hsiao M., Chang Y.-C., Hung S.-C. (2019). Ambient particulate matter attenuates Sirtuin1 and augments SREBP1-PIR axis to induce human pulmonary fibroblast inflammation: Molecular mechanism of microenvironment associated with COPD. Aging.

[58] Hamra G.B., Guha N., Cohen A., Laden F., Raaschou-Nielsen O., Samet J.M., Vineis P., Forastiere F., Saldiva P., Yorifuji T. (2014). Outdoor Particulate Matter Exposure and Lung Cancer: A Systematic Review and Meta-Analysis. Environ. Health Perspect..

[59] Yokoi S., Yasui K., Iizasa T., Imoto I., Fujisawa T., Inazawa J. (2003). TERC identified as a probable target within the 3q26 amplicon that is detected frequently in non-small cell lung cancers. Clin. Cancer Res..

[60] Wu X., Ruan L., Yang Y., Mei Q. (2016). Identification of crucial regulatory relationships between long non-coding RNAs and protein-coding genes in lung squamous cell carcinoma. Mol. Cell. Probes.

[61] Arbyn M., Weiderpass E., Bruni L., De Sanjosé S., Saraiya M., Ferlay J., Bray F. (2020). Estimates of incidence and mortality of cervical cancer in 2018: A worldwide analysis. Lancet Glob. Health.

[62] Iarc W.G. (2007). Human papillomaviruses. IARC Monogr. Eval. Carcinog. Risks Hum..

[63] Carrillo D., Muñoz J.P., Huerta H., Leal G., Corvalan A.H., León O., Calaf G.M., Urzúa U., Boccardo E., Tapia J.C. (2017). Upregulation of PIR gene expression induced by human papillomavirus E6 and E7 in epithelial oral and cervical cells. Open Biol..

[64] Komai K., Niwa Y., Sasazawa Y., Simizu S. (2015). Pirin regulates epithelial to mesenchymal transition independently of Bcl3-Slug signaling. FEBS Lett..

[65] Tabrizi R., Vakili S., Akbari M., Mirhosseini N., Lankarani K.B., Rahimi M., Mobini M., Jafarnejad S., Vahedpoor Z., Asemi Z. (2019). The effects of curcumin-containing supplements on biomarkers of inflammation and oxidative stress: A systematic review and meta-analysis of randomized controlled trials. Phytother. Res..

[66] Calaf G.M., Urzúa U., Termini L., Aguayo F. (2018). Oxidative stress in female cancers. Oncotarget.

[67] Thacker P.C., Karunagaran D. (2015). Curcumin and Emodin Down-Regulate TGF-β Signaling Pathway in Human Cervical Cancer Cells. PLoS ONE.

[68] Gallardo M., Calaf G.M. (2016). Curcumin inhibits invasive capabilities through epithelial mesenchymal transition in breast cancer cell lines. Int. J. Oncol..

[69] Gallardo M., Kemmerling U., Aguayo F., Bleak T.C., Calaf G.M. (2019). Curcumin rescues breast cells from epithelial-mesenchymal transition and invasion induced by anti-miR-34a. Int. J. Oncol..

[70] Aedo-Aguilera V., Carrillo-Beltrán D., Calaf G.M., Muñoz J.P., Guerrero N., Osorio J.C., Tapia J.C., León O., Contreras H.R., Aguayo F. (2019). Curcumin decreases epithelial-mesenchymal transition by a Pirin-dependent mechanism in cervical cancer cells. Oncol. Rep..

[71] Brzóska K., Stępkowski T.M., Kruszewski M. (2014). Basal PIR expression in HeLa cells is driven by NRF2 via evolutionary conserved antioxidant response element. Mol. Cell. Biochem..

[72] Rogers H.W., Weinstock M.A., Feldman S.R., Coldiron B.M. (2015). Incidence Estimate of Nonmelanoma Skin Cancer (Keratinocyte Carcinomas) in the US Population, 2012. JAMA Dermatol..

[73] Siegel R.L., Miller K.D., Jemal A. (2020). Cancer statistics, 2020. CA Cancer J. Clin..

[74] Licciulli S., Luise C., Zanardi A., Giorgetti L., Viale G., Lanfrancone L., Carbone R., Alcalay M. (2010). Pirin delocalization in melanoma progression identified by high content immuno-detection based approaches. BMC Cell Biol..

[75] Licciulli S., Luise C., Scafetta G., Capra M., Giardina G., Nuciforo P., Bosari S., Viale G., Mazzarol G., Tonelli C. (2011). Pirin Inhibits Cellular Senescence in Melanocytic Cells. Am. J. Pathol..

[76] Siegel R.L., Miller K.D., Jemal A. (2019). Cancer statistics, 2019. CA Cancer J. Clin..

[77] Hirota A., Kawachi Y., Itoh K., Nakamura Y., Xu X., Banno T., Takahashi T., Yamamoto M., Otsuka F. (2005). Ultraviolet A Irradiation Induces NF-E2-Related Factor 2 Activation in Dermal Fibroblasts: Protective Role in UVA-Induced Apoptosis. J. Investig. Dermatol..

[78] Sample A., Zhao B., Wu C., Qian S., Shi X., Aplin A., He Y.-Y. (2018). The Autophagy Receptor Adaptor p62 is Up-regulated by UVA Radiation in Melanocytes and in Melanoma Cells. Photochem. Photobiol..

[79] Thomsen K.G., Terp M.G., Lund R.R., Søkilde R., Elias D., Bak M., Litman T., Beck H.C., Lyng M.B., Ditzel H.J. (2015). miR-155, identified as anti-metastatic by global miRNA profiling of a metastasis model, inhibits cancer cell extravasation and colonization in vivo and causes significant signaling alterations. Oncotarget.

[80] Xiang X., Zhuang X., Ju S., Zhang S., Jiang H., Mu J., Zhang L., Miller D., Grizzle W., Zhang H.-G. (2011). miR-155 promotes macroscopic tumor formation yet inhibits tumor dissemination from mammary fat pads to the lung by preventing EMT. Oncogene.

[81] Higashi K., Tomigahara Y., Shiraki H., Miyata K., Mikami T., Kimura T., Moro T., Inagaki Y., Kaneko H. (2011). A Novel Small Compound That Promotes Nuclear Translocation of YB-1 Ameliorates Experimental Hepatic Fibrosis in Mice. J. Biol. Chem..

[82] Yamaoka H., Sumiyoshi H., Higashi K., Nakao S., Minakawa K., Sumida K., Saito K., Ikoma N., Mabuchi T., Ozawa A. (2014). A novel small compound accelerates dermal wound healing by modifying infiltration, proliferation and migration of distinct cellular components in mice. J. Dermatol. Sci..

[83] Cheeseman M.D., Chessum N.E.A., Rye C.S., Pasqua A.E., Tucker M.J., Wilding B., Evans L.E., Lepri S., Richards M., Sharp S.Y. (2016). Discovery of a Chemical Probe Bisamide (CCT251236): An Orally Bioavailable Efficacious Pirin Ligand from a Heat Shock Transcription Factor 1 (HSF1) Phenotypic Screen. J. Med. Chem..

[84] DeSantis C.E., Ma J., Bryan L., Jemal A. (2013). Breast cancer statistics, 2013. CA Cancer J. Clin..

[85] Perou C.M., Sørlie T., Eisen M.B., Van De Rijn M., Jeffrey S.S., Rees C.A., Pollack J.R., Ross D.T., Johnsen H., Akslen L.A. (2000). Molecular portraits of human breast tumours. Nature.

[86] Veer L.J.V., Dai H., Van De Vijver M.J., He Y.D., Hart A.A.M., Mao M., Peterse H.L., Van Der Kooy K., Marton M.J., Witteveen A.T. (2002). Gene expression profiling predicts clinical outcome of breast cancer. Nature.

[87] Karlsson E., Delle U., Danielsson A., Olsson B., Abel F., Karlsson P., Helou K. (2008). Gene expression variation to predict 10-year survival in lymph-node-negative breast cancer. BMC Cancer.

[88] Shubbar E., Helou K., Kovács A., Nemes S., Hajizadeh S., Enerbäck C., Einbeigi Z. (2013). High levels of γ-glutamyl hydrolase (GGH) are associated with poor prognosis and unfavorable clinical outcomes in invasive breast cancer. BMC Cancer.

[89] Suleman M., Chen A., Ma H., Wen S., Zhao W., Lin D., Wu G., Li Q. (2019). PIR promotes tumorigenesis of breast cancer by upregulating cell cycle activator E2F1. Cell Cycle.

[90] DeGregori J. (2002). The genetics of the E2F family of transcription factors: Shared functions and unique roles. Biochim. Biophys. Acta.

[91] Sun C.C., Li S.-J., Hu W.-D., Zhang J., Zhou Q., Liu C., Li L.-L., Songyang Y.-Y., Zhang F., Chen Z.-L. (2019). Comprehensive Analysis of the Expression and Prognosis for E2Fs in Human Breast Cancer. Mol. Ther..

[92] Hutter C., Zenklusen J.C. (2018). The Cancer Genome Atlas: Creating Lasting Value beyond Its Data. Cell.

[93] Bergman A.-C., Alaiya A.A., Wendler W., Binétruy B., Shoshan M., Sakaguchi K., Bergman T., Kronenwett U., Auer G., Appella E. (1999). Protein kinase-dependent overexpression of the nuclear protein pirin in c-JUN and RAS transformed fibroblasts. Cell. Mol. Life Sci..

[94] Yi Y.W., Oh S. (2015). Comparative analysis of NRF2-responsive gene expression in AcPC-1 pancreatic cancer cell line. Genes Genom..

[95] Liu J., Liu W., Li H., Deng Q., Yang M., Li X., Liang Z. (2019). Identification of key genes and pathways associated with cholangiocarcinoma development based on weighted gene correlation network analysis. PeerJ.

[96] Goldman M., Craft B., Brooks A., Zhu J., Haussler D. (2019). The UCSC Xena platform for public and private cancer genomics data visualization and interpretation. BioRxiv.

[97] Panchal H.D., Vranizan K., Lee C.Y., Ho J., Ngai J., Timiras P.S. (2008). Early Anti-Oxidative and Anti-Proliferative Curcumin Effects on Neuroglioma Cells Suggest Therapeutic Targets. Neurochem. Res..

[98] Edwards H., Rubenstein M., Dombkowski A.A., Caldwell J.T., Chu R., Xavier A.C., Thummel R., Neely M., Matherly L.H., Ge Y. (2016). Gene Signature of High White Blood Cell Count in B-Precursor Acute Lymphoblastic Leukemia. PLoS ONE.

[99] Teachey D.T., Hunger S.P. (2013). Predicting relapse risk in childhood acute lymphoblastic leukaemia. Br. J. Haematol..

